# Ocular manifestations of people living with HIV in Tunisia

**DOI:** 10.4102/sajhivmed.v22i1.1193

**Published:** 2021-03-19

**Authors:** Dorsaf Saadouli, Lamia Ammari, Khaoula Ben Mansour, Yosra Yahyaoui, Sameh Aissa, El Afrit Mohamed Ali, Salem Yahyaoui, Hanene Tiouri

**Affiliations:** 1 Department of Ophthalmology, La Rabta Hospital, Faculty of Medicine of Tunis, University of Tunis El Manar, Tunis, Tunisia; 2 Department of Infectious Diseases, La Rabta Hospital, Faculty of Medicine of Tunis, University of Tunis El Manar, Tunis, Tunisia; 3 Faculty of Medicine of Tunis, University Tunis El Manar, Tunis, Tunisia

**Keywords:** acquired immunodeficiency syndrome, human immunodeficiency virus, ocular manifestation, uveitis, immune recovery uveitis, cytomegalovirus retinitis

## Abstract

**Background:**

Ocular involvement is a common complication of human immunodeficiency virus (HIV). Knowledge about this topic in Tunisia is limited.

**Objective:**

To investigate ophthalmic manifestations in patients living with HIV in Tunisia.

**Method:**

This was an observational study, performed between January 2007 and December 2016. We included patients with ocular disorders related to HIV. The data were recorded retrospectively from chart review.

**Results:**

Amongst 98 people living with HIV (PLWH), 36 participants (55 eyes) had ocular manifestations. The mean age was 32.2 ± 5.6 years. Twenty-four patients were men and 12 were women. The mean value of CD4+ T-cell count was 156.5 ± 4.2 cells/µL. Bilateral lesions were found in 19 eyes. Best corrected visual acuity was better than 6/12 in 36 eyes. The most common ocular finding was dry eye syndrome (22%), cotton-wool spots (20%) and retinal haemorrhage (16%) followed by cytomegalovirus (CMV) retinitis (9%), anterior uveitis (7%), toxoplasmosis (4%) and tuberculosis retinochoroiditis (7%) Herpetic keratitis (5%), Herpes zoster ophthalmicus (2%) and syphilitic chorioretinitis (2%). Papilledema was found in three eyes (5%). Panuveitis was observed in four eyes (7%): three of them were associated with chorioretinal toxoplasmosis, syphilitic chorioretinitis and CMV retinitis. The fourth was attributable to immune recovery uveitis. A CD4+ T-cell count of ≤ 200 cells/µL was found to be an independent risk factor for developing posterior segment manifestations.

**Conclusion:**

Various ophthalmic manifestations were observed in PLWH. The most common lesion was retinopathy. Ocular involvement can be serious leading to poor visual prognosis, which requires close collaboration between the ophthalmologist and infectious disease physician.

## Introduction

Human immunodeficiency virus (HIV) is a major global public health problem.^1^

Immunodeficiency caused by the virus results in increased susceptibility to opportunistic infections and neoplasms.^2^ By increasing access to antiretroviral drugs, this disorder changed from a high-mortality disease to a manageable chronic disease, which may affect every organ system.^3^ In Tunisia, HIV prevalence was 0.016% and patients were mainly infected with HIV-1.^4,5^

Ocular complications often reflect systemic disease and have become more common and occur in approximately 70% – 80% of people living with HIV (PLWH).^1^ It has been shown that the prevalence of ocular problems in PLWH is higher than in uninfected patients.^6,7^ However, the clinical features of these ocular manifestations differ between countries, especially between high-income and low to middle-income countries.^8^ This is because of improved accessibility to healthcare and to antiretroviral therapy (ART), which has shifted the spectrum of the disease from infectious to non-infectious affections.

Most ocular manifestations respond well to treatment, so it is important to recognise them in order to establish an early diagnosis and to start an adequate management programme. Knowledge about this topic in Tunisia, a North African country, is limited.^9^ Here, we aimed to investigate ophthalmic manifestations in PLWH in the Tunisian population.

## Methods

This retrospective study was carried out in the ophthalmology department of La Rabta hospital of Tunis over a period of 10 years between January 2007 and December 2016. The median year that most of the participants presented (9 patients) was 2013. It included patients with ocular disorders related to HIV. Patients were referred from the department of infectious diseases, which is an HIV reference centre.

Human immunodeficiency virus classification was determined according to the Centers for Disease Control and Prevention (CDC) and World Health Organization (WHO) Staging Systems.^2,10^ Patients with other causes of immunodeficiency and those with additional medical problems that may overlap with ocular manifestations were excluded from our study.

Data extracted retrospectively from the chart reviews, included: age, gender, corrected visual acuity (VA) (measured with a Snellen chart), anterior segment slit-lamp examination, intraocular pressure (IOP) with Goldmann applanation tonometry, dilated fundus examination and fluorescein angiography if appropriate.

Antiretoviral treatment status and CD4+ T-cell counts were also collected.

The diagnosis of cytomegalovirus (CMV) retinitis (fulminant or indolent form) was based on clinical characteristics of a necrotic retinitis with irregular borders with or without haemorrhage and small adjacent dot-like, white satellite lesions.^8,11^

Immune recovery uveitis was diagnosed when non-infectious uveitis occurred in patients with inactive CMV retinitis who showed a substantial elevation in CD4+ T-cell count by 50 cells/µL or more to a level of 100 cells/µL or more with highly active antiretroviral therapy (HAART).^11^

All statistical analyses were performed using SPSS 20. Comparison of continuous data was performed with the independent samples of Student’s-*t* test, the Mann–Whitney test or the Wilcoxon signed-rank test. Categorical data were analysed with the chi-squared test or the Fisher exact test. We conducted a logistic regression analysis in descending order, to identify factors independently associated with posterior segment involvement. Statistical significance was defined as *p* < 0.05.

### Ethical consideration

Ethical approval was obtained from Stellenbosch University Health Research Ethics Committee, reference number: S19/06/109.

## Results

Of the 98 patients living with HIV who underwent an ophthalmic examination, 36 cases (55 eyes) (36.7%.) had ocular manifestations (Table 1). Twenty-four patients were men and 12 were women, the sex ratio was 2. The age of the patients with ocular manifestations ranged from 18 to 56 years with mean age of 32.2 ± 5.6 years (Table 2). Seventy per cent of patients were in the age group of 30–39 years. Most patients (86%) were on ART. Tenofovir + emtricitabine + efavirenz combination-therapy has been prescribed since 2011. However, before this period patients were treated with individual drugs, zidovudine + lamivudine and efavirenz. Those who did not receive ART were: one new case with first diagnosis, poor compliance with treatment because of depression (1 patient) and tuberculosis infection, which resulted in the delay of the antiretroviral therapy (3 patients). Systemic opportunistic infections at the time of ophthalmic examination were noted in six patients. They included pulmonary tuberculosis (three patients), pneumocystis pneumonia (two patients), cryptococcal meningitis (one patient) and candidiasis (one patient). Fifty-five per cent of cases were in the WHO Clinical Stage 3 of HIV disease. Of these, five patients did not receive antiretroviral therapy.

**TABLE 1:** Ocular manifestations in patients living with HIV (*n* = 36).

Ocular manifestations	Percentage
Dry eye syndrome	22.0
Herpetic keratitis	5.4
Herpes zoster ophthalmicus	2.0
Anterior uveitis	7.0
Cotton-wool spots	20.0
Intra-retinal hemorrhages	16.0
Toxoplasmosis retinochoroiditis	4.0
Tuberculosis retinochoroiditis	7.0
Syphilitic chorioretinitis	2.0
CMV retinitis	9.0

CMV, cytomegalovirus.

**TABLE 2:** Baseline demographic characteristics of the study participants listed.

Variables	Participants with HIV-related ocular manifestations (*N* = 36)			Participants without HIV-related ocular manifestations (*N* = 62)			*P*
	Mean ± SD	Patients	%	Mean ± SD	Patients	%	
**Age (years)**	32.2 ± 5.6	-	-	34.4 ± 1.9	-	-	0.070
< 20 years	-	2	-	-	4	-	-
20-29 years	-	4	-	-	11	-	-
30-39 years	-	25	-	-	34	-	-
40-49 years	-	3	-	-	9	-	-
> 50 years	-	2	-	-	4	-	-
**Sex**							
Male	-	24	-	-	38	-	-
Female	-	12	-	-	24	-	-
**Patients receiving ART**	-	31	86	-	58	93	0.200
**Mean CD4+ T cell count (cells/μL) ± SD**	156.5 ± 4.2	-	-	264 ± 4.3	-	-	0.001
**Opportunistic systemic infections**	-	6	16	-	4	6.4	0.040
**WHO Clinical Stage 3 of HIV disease.**	-	20	53	-	10	16	0.060

HIV, human immunodeficiency virus; SD, standard deviation; ART, antiretroviral therapy; WHO, World Health Organization.

Slightly more than half of the patients (55%) had CD4+ T-cell counts < 200 cells/µL. The mean value of the CD4+ T-cell count was 156.5 (SD ± 4.2 cells/µL); the median value was 172 cells/µL, range: 23–359 cells/µL. The four cases with CMV retinitis had CD4+ T-cell counts below 50 cells/µL, with a mean value of 36.4 ± 5.2 cells/µL.

Various ocular manifestations were noted. Bilateral lesions were found in 19 eyes. Twenty-nine of the eyes (53%) had a best corrected visual acuity (BCVA) better than or equal to 6/12, whilst 11 eyes (20%) had a BCVA between 6/60 and 6/12 and 15 eyes (27%) had a BCVA acuity lower than 6/60. The main manifestations were decreased and/or blurred vision (20 eyes) followed by redness (eight eyes) and floaters (five eyes). Nine cases (11 eyes) did not show this manifestation.

Anterior segment manifestations were noted in 36.4% of cases. Most were not specific: dry eye syndrome found in 12 eyes (22%), and complicated by bilateral corneal ulcer in one. Others were opportunistic viral infections: Herpes keratitis in three eyes: (5.4%) and Herpes zoster ‘ophthalmicus’ in one (2%).

Anterior uveitis was found in four eyes (7%), which was attributed to the toxic effects of the HIV protease inhibitor Indinavir in all cases.

Posterior segment involvement was seen in 32 eyes of 23 patients (58%). The most common manifestation was retinal microvasculopathy observed in 20 eyes (36%) of 13 patients. The manifestations were characterised by cotton-wool spots noted in 11 eyes (20%) and intra-retinal haemorrhages noted in nine eyes (16%). The other posterior segment manifestations included toxoplasmosis in 2 (4%) and tuberculosis retinochoroiditis in 7 (9%) eyes, syphilitic chorioretinitis in one eye (2%) and CMV retinitis involving five eyes (9%). Panuveitis was observed in four eyes (7%): three with chorioretinal toxoplasmosis, syphilitic chorioretinitis and CMV retinitis each. The fourth had a paradoxical worsening of intraocular inflammation after starting ART, which was attributed to immune recovery uveitis. The neuro-ophthalmic manifestations observed included bilateral disc oedema secondary to cryptococcal meningitis in one patient and unilateral optic neuropathy in one patient.

The ocular manifestations observed in the five patients who did not receive ART were as follows: tuberculosis retinochoroiditis (3 cases) and CMV retinitis (2 cases).

A CD4+ T-cell count of < 200 cells/µL was found to be an independent risk factor for developing posterior segment disease, adjusted OR = 3.2, CI 95%, 1.1–3.2, *p* = 0.01. However, the number of samples was too small to do a comparison of male versus female and ART treated versus ART-naïve patients.

## Discussion

During the course of the disease, the prevalence of ophthalmic manifestations in PLWH ranged from 19% to 70%.^2,12^ It differs by geographic region, which is related to regional HIV prevalence.^12^ Although it has been considerably reduced by the introduction of ART, it may still be high, especially in areas with high HIV prevalence such as sub-Saharan Africa.^12,13,14^ This may be because of an increase in the survival of these patients.^12,13,14^ In the present study, the prevalence of ocular involvement was 36.7%, which is comparable to other African studies.^13,15^ However, ocular manifestations in this study might be under-reported because most of the PLWH do not receive routine ophthalmic screening.

Previous studies have found demographic patterns similar to that of our study, such as male preponderance likely related to the greater risk-taking sexual behaviour of men.^16^ In addition, our most susceptible age group with ocular complications of HIV, namely those in their 30s, was also described in a Ghanaian study.^17^

The ocular manifestations are varied. Almost all the structures of the eye may be affected. It has been demonstrated that the CD4+ T-cell count may help to predict the occurrence of ocular manifestations in PLWH. Indeed, the risk of developing ocular disease in PLWH is increased when the CD4 count is < 200 cells/µL.^2,13^ This is consistent with our findings. Although sufficient care is generally provided in our country, patients often do not adhere to treatment and to follow-up. This may explain the relatively low value of the mean CD4+ T-cell count in the present study. Moreover, based on a French study comparing the clinical features of North African and French PLWH, the difference concerned only the adherence to care, which could be related to a problem of acceptability of the disease.^7^

The ocular manifestations may be classified as opportunistic infections, retinal microangiopathy, neoplasms, neuro-ophthalmic lesions and immune reconstitution inflammatory syndrome (IRIS).^18^ Antiretroviral therapy has improved the length and quality of life by decreasing opportunistic infections.^19^

Consequently, the prevalence of anterior segment complications and adnexal disorders compared with posterior segment manifestations has increased.^19^ Its prevalence ranged from 7% to 33.9%.^18,19,20^

These disorders were 36.4% in the present study.

Anterior segment disorders can lead to ocular morbidity and so affect the quality of life of patients. Dry eye, blepharitis and uveitis are the most common anterior segment complications. This finding is consistent with our study that noted a predominance of dry eye syndrome (22%), possibly contributed to, by the region’s climate. Ophthalmic herpes zoster, noted in one eye in our series, can be considered a possible indicator of HIV-infection, especially in young adults (< 50 years of age).^21^ Moreover, it has been shown that anterior uveitis was less frequent than posterior uveitis.^18^ Drug induced and immune recovery uveitis and herpetic uveitis were the main causes reported by Singalavanija.^18^ In our study anterior uveitis was related to Indinavir therapy in all cases. The low prevalence of anterior uveitis mentioned in the present study (7%) suggests a delayed consultation, which could lead to a serious issue. Neoplasms of the eyelid or the conjunctiva, in PLWH are not rare. Kaposi’s sarcoma may affect up to 10%.^19^ However, we did not encounter anyone with this neoplasm.^19^

As reported in previous African data the most common ocular involvement in PLWH was retinopathy that is characterised by cotton-wool spots or associated with intra-retinal haemorrhages.^14,15,22^ Patients having such manifestations are usually asymptomatic.^14,15^

Posterior segment manifestations include, in addition to retinopathy, opportunistic infections of the retina and the choroid. The introduction of ART has led to a decrease in the incidence of these disorders.^7,21^ Various opportunistic pathogens can be associated with HIV infection. Cytomegalovirus retinitis was the most common ocular opportunistic infection and was most often associated with CD4+ T-cell count < 50 cells/µL. However, ocular syphilis is considered as undiscriminating and does not associate with CD4+ T-cell count. Several studies showed that panuveitis is the most common ocular presentation in PLWH.^21,23,24,25^ This is consistent with the clinical presentation of our only patient who presented with syphilitic panuveitis.

The results of this study are similar to the findings of other African studies. Although the incidence of ocular complications in HIV infection has declined since the advent of ART, it remains the main cause of visual loss in patients with AIDS in developing countries because of limited medical resources, which can lead to a poorer systemic management. In the present study, 27% of patients had a BCVA < 6/60. So the management of such patients represents a macro-issue of public health in our country. The early detection of ocular involvement in PLWH is highly recommended in order to prevent severe vision loss or at least minimise it.

The limitation of our retrospective study is the internal validity because of the selection bias. In addition, male predominance may be a possible bias. We propose a prospective study with a larger sample size with systematic screening examinations, comparing uninfected people with ophthalmic disorders to PLWH. This would show if there is an increase in cases or severity because of HIV.

## Conclusion

Ophthalmic manifestations in Tunisian PLWH are varied. The most common lesion was retinopathy. However, ocular involvement in PLWH can be serious leading to poor visual prognosis, especially in developing countries. Although the introduction of ART has decreased the incidence and the severity of opportunistic ocular infections, newer manifestations such as drug induced and immune recovery uveitis have emerged as a cause of visual loss. Therefore, the collaboration between ophthalmologists and infectious disease physicians is strongly recommended in the diagnosis and management of these patients.
